# Nutrition in the Elderly According to Traditional Persian Medicine

**DOI:** 10.31661/gmj.v8i0.1138

**Published:** 2019-05-09

**Authors:** Hossein Rezaeizadeh, Maryam Abbasi

**Affiliations:** ^1^Faculty Persian Medicine, School of Parsian Medicine, Tehran University of Medical Sciences, Tehran, Iran

**Keywords:** Nutrition, Aging, Elderly, Traditional Persian Medicine

## Dear Editor,

As we know, due to increased life expectancy, the world’spopulation is aging,and Iran deals with the same challenge. Since population aging may lead to increased prevalence of malnutrition [1]; particular focus is required for the prevention and treatment of malnourished elderly. Our materials were selected by searching tutorials of Traditional Persian medicine (TPM) in TPM old books such as The Canon of Medicine (*Al-Kanoon Fi Al-Tib*) and *KholasatOl-Hikmah* about lifestyle management in the elderly. In old Persian medicine books, special attention has been given on aging, and the importance of nutrition and other aspects of elderly life have been explained in special parts in order to promote quality of life for elderly [2, 3]. TPM scholars emphasized that due to the weakness of the gastrointestinal (GI) system especially the stomach in the elderly, they should eat more often than young adults but in smaller portions. It is very important for elderly to eat dinner and not to sleep hungry, as skipping dinner can lead to body weakness that cannot be compensated easily; dinner should be eaten in the evening and not late [2, 3]. TPM scholars mentioned some useful foods and vegetables for the elderly. They have recommended the use of well-cooked wheat bread, soft-boiled eggs, poultry soups and pea-soup with almond or olive oil [4]. Regarding that constipation is one of the most common complaints in elderly that can accompany with slow gastric emptying and small bowel transit [5], treatment of constipation is important in order to improve the appetite [6], nutraceutical agents, dietary supplements, that help to alleviate constipation, can have a remarkable role in dissolving malnutrition in elderly. They should eat vegetables such as beet leaves or parsley and some leek cooked with olive oil before their meal to prevent constipation. The best fruits for the elderly are fig and then grape; fig can be taken fresh with or without prune in summer or dried and cooked with honey in winter [2]. Consumption of fig with walnut has also been advised for the elderly. Fig is one of the best for relieving constipation [4]; moreover, fig had good effects on memory in animal studies [7]. Eating lettuce before sleep can help the elderly to have a better sleep, but it should be taken with warm spices [3]. The milk can be also beneficial for the elderly if their GI system can tolerate it [2]. Some foods such as eggplant, lentil, cow meat and old meats, sour and acidic materials such as vinegar and pickles, watermelon, cucumber, squash, and ice-cold water should be avoided for routine use in elderly according to TPM texts [4]. We suggest well-designed clinical trials evaluate the effects of recommended foods in the elderly and eventually using them for improving the elderly’s health.

## Acknowledgment

We appreciate Doctor Fahimeh Habibi for editing and English revision of this letter.

## Conflicts of Interest

The authors have no conflicts of interest in writing this article.
